# Rank of Medical Officers in the Navy

**Published:** 1851-03

**Authors:** 


THE MEDICAL EXAMINER.

PHILADELPHIA, MARCH, 1851.

### RANK OF MEDICAL OFFICERS IN THE NAVY.

Our readers were informed in November last, that a board of of captains was convened to consider the subject of military rank in the navy, in all its various relations. Their report was submitted to Congress on the 21st January, 1851. The board was composed of officers of the line of the navy exclusively; no one of the staff corps was represented directly or indirectly. A surgeon was ordered to appear before it and advocate the claims of medical officers to have assigned them a rank, that is, a definite position in the military organization of which they are members. The same duty was also assigned to a purser. Both the gentlemen were released from the order at their own request. It is understood that, though willing to sit as members, they declined to appear as advocates before a board, the functions of which were limited to expressing an opinion; because as advocates they could have no vote in forming that joint opinion, or of presenting a minority report, should circumstances require it. The board being constituted of gentlemen whose opinions were formed, probably many years before they were called upon to express them officially, it would be no easy task to change their views on the subject in any essential particular. While it is cheerfully conceded that their opinions are sincerely entertained, it is firmly believed they are erroneous to some extent.

To balance or off-set the overbearing influence which the arguments of two staff officers might exert upon the deliberations of the board, two lieutenants of the line were ordered to advocate the claims of the line, or in a word, to satisfy the board that staff officers in the navy should not have any rank. The line advocates performed their duty before the line officers without opposition; and thus the board was left to make up its judgment unembarrassed by any necessity of weighing conflicting testimony and opinions prior to arriving at a conclusion.

Considering the constitution of the board, the presumed class-feeling of opposition to the staff on this point—and it should be distinctly understood the opposition extends no further—and the fixed mental habit of relying upon precedent, the report is quite as liberal as could have been reasonably anticipated.

Substantially the Board recommends first, the creation of new grades of officers in the naval service, namely :—

A grade of line officers, under the title of lieutenant commandant, to occupy a position between the present grades of commander and lieutenant, whose duty would be to command brigs and smaller vessels. The board is of opinion that the addition of such a grade would render the navy more efficient, and seemingly assign, as the only argument for adding another grade of commanding officers to the service, that "it was known during the revolution and prior to the act of March 3, 1801."

It is suggested that the grade of master, heretofore appointed by warrant, shall hereafter be commissioned, because they have "always been associated with commission officers, and their duties require more varied and much higher attainments than are necessary for several other grades of navy officers who now have commissions." The most prominent duties of a master in the navy are to navigate the vessel, and to control the stowage of provisions, &c., in the hold.

It is suggested that the grade of passed-midshipmen should be recognized by law, although the board seems to be unable or unwilling to assign to the grade, duties which are distinct, or in any manner separate from those of midshipmen who have not passed an examination, for promotion to the grades of master and lieutenant.

The board refrained from recommending grades of admirals, being content merely to allude to the subject; because, "motives which must be obvious, when the composition of the board is considered, prevented it from offering any suggestions beyond the limits fixed by the law."

The board recommends that passed assistant surgeons should constitute a distinct grade of medical officers, but does not suggest for it any duties different from those now assigned.

A grade of surgeons, to be denominated "Hospital Surgeons," whose duties would be limited to serving at sea as surgeons of the fleet, and in hospitals on shore is also recommended. The requisite to appointment in this grade is twenty years service in the medical corps or staff of the navy.

It is suggested that a grade of assistant pursers, to be appointed by warrant, should be created.

The second prominent feature of the board's report, is that the "order of subordination may be expressed in the navy by the separate and combined use of the terms *rank*, *precedence*, and *command*, employed technically."

The word *rank* is to be limited, confined exclusively in its application to officers of the line of the navy—that is, " sea-officers." The proposition of the board is to confine " the use of the term *rank* strictly to those officers who may be eligible to exercise *command*, and only in connexion with, and to the extent of that eligibility; to apply in the same manner the term *precedence* to officers who are not eligible to exercise *any command*, or whose eligibility is limited to their own corps, or when used in comparing the official relations between officers whose relative positions are designated by rank, and those whose positions are regulated by precedence."

The word precedence is thus substituted for the well understood term, assimilated rank; and it is feared the effect of this substitution will be to impose upon all staff officers of the navy the condition of a *caste*, in all things inferior to the line, and be thus made to hold a footing among " sea-officers," similar to that of Jews in a community of strict Roman Catholics; or, at least, similar to that of free citizens of a republic legally deprived of the right of suffrage. If the term precedence be adopted, it is clear, medical officers will have no rank, no military position properly so called.

The word *rank*, applied in military organization, means nothing more nor less than the position of one officer relatively to all other officers. The improper use of the terms *rank* and *grade* synonymously, tends to confound the true meaning of the former. There may be many officers of the same *grade*; all captains are of the same grade, but according to legitimate use of the words, two officers, although of the same grade, cannot be of the *same rank*, that is, possess the same identical official position. Rank determines precedence, and, when on duty, the right of an officer to exercise authority over those below him, as well as his obligation to obey those above him in his grade. Rank in itself confers no authority whatever, but simply renders the officer eligible to exercise the kind and degree of authority described in his commission, either as a superior or inferior of those associated with him on duty.

If the position of a hospital surgeon be designated by the term of precedence, it might be questionable in the minds of casuists in the line of the navy, whether the surgeon had any right to command even a midshipman while in a hospital—to control his going out or coming in—in a word, to exact obedience to his reasonable and legitimate commands as a surgeon by military laws and tribunals. According to the spirit and letter of the proposed law, a staff officer on duty never can be superior to any officer of the line, therefore, the fault of " disrespect to his superior officer " never can be charged by him upon the latter, even under the most flagrant circumstances.

It is doubtful if legislation can make "precedence" a synonym of rank or assimilated rank. It should not be forgotten, in arguments upon subjects like the present, that laws are requisite only for refractory, unreasonable, or weak-minded men: and that none but military laws can apply to members of a military organization.

One more remark on the use of terms: the term "civil officer" is not applicable to any officer subject to punishment by decision of a military court: the use of it in the navy tends to make *caste* distinctions. The term "sea officers," is not an accurate verbal designation of officers of the line, for it is rational to include in the term all officers who serve at sea, without regard to their specific official duties. It is desirable to avoid the use of designations or terms which tend to make caste distinctions in a community of gentlemen equally entitled to consideration. It is supposed by many, whether correctly or not is unimportant, that military men are prone to regard themselves, as a class, superior to simple citizens, or even to those holding civil offices, and for this reason, the term staff being in itself technically correct, should be used in law to designate those who have been heretofore called "civil officers" in the navy. It is desirable that no official designations should be employed, calculated to sustain notions of personal superiority or inferiority, in a community having a general object, the attainment of which depends somewhat on a harmony of parts. Naval efficiency cannot be easily attained without a cheerful co-operation of all members of the service. It is time the conflicting opinions on the subject of rank were settled on some fair compromise.

These preliminary observations will enable our readers to appreciate justly the position proposed for medical officers in the navy. The board divides the staff officers into six classes, and assigns each class a position relatively to some one of the six subordinate grades of officers of the line, as follows:—

| Staff Officers. [ Miscalled "Civil Officers." ] | Line Officers. [ Miscalled "Sea Officers." ] |
|---|---|
| *First class.* | |
| Hospital Surgeons, [if authorized.] | Shall have precedence with, but after *lieutenants commandant.* |
| *Second class.* | |
| Engineer in Chief,<br>Chaplains,<br>Surgeons. | With *lieutenants,* and with each other, according to the date of their respective commissions. |

| STAFF OFFICERS. [Miscalled "Civil Officers."] | LINE OFFICERS. [Miscalled "Sea Officers."] |
|---|---|
| *Third class.* | |
| Chief Engineers, Passed Assistant Surgeons, Professors of Mathematics, Secretaries to Commanders of squadrons. | With, but after *masters.* |
| *Fourth class.* | |
| Assistant Surgeons, Assistant Pursers [if authorized,] First Assistant Engineers. | With, but after *passed midshipmen.* |
| *Fifth class.* | |
| Second Assistant Engineers, Clerks to Commanders of Squadrons, Clerks to Commanding Officers, Clerks to Pursers. | With, but after *midshipmen.* |
| *Sixth class.* | |
| Third Assistant Engineers. | With, but after *boatswains.* |

The "precedence" of the several classes and grades of staff officers relatively to each other, is according to the order in which they are named in the above scale; except secretaries and clerks, who shall have precedence "according to the rank of the officers to whom they may be attached or allowed."

Except in the second class, all staff officers are placed in the above arrangement so that they shall ever be the junior, or rather the youngest of the lineal grade with which they are assimilated in rank. An assistant surgeon, for example, after any period of service, even five or six years, must yield precedence to the youngest passed midshipman on the list. Midshipmen are cadets or professional pupils; passed midshipmen are those who, upon examination, have been found eligible for promotion, but are still required to discharge the duties of midshipmen, until vacancies above enable them to advance to the higher grades.

Passed assistant surgeons are made thus the permanent juniors of the grade of master.

Surgeons are assimilated in rank with lieutenants, according to date of commission; and hospital surgeons are made the permanent juniors of the lowest grade of commanding officers of the line, yet to be created. The proposed law requires that a surgeon shall not become hospital surgeon until he has been twenty years commissioned in the medical corps but there is no *time* qualification proposed for the appointment of lieutenants commanding, and if they reach the grade after being commissioned for five years in the navy, which may be possible under particular circumstances, they have precedence of the oldest hospital surgeon.

But the board has thought that even the nominal position given to medical and other staff officers in this scale might be excessive. It has taken away even the name of rank, and, while dictating a military law to govern them, denominated them "civil officers." Still fearing the aspirations of the latter to equality of social and professional rights and privileges under this scale, might embarrass the line officers, the board has provided for a suspension of the operation of assimilated rank in most cases in which it should be operative. Lineal rank is held to be inviolable both by the executive and legislative departments of the government; but assimilated rank is of such a pliant nature that it must yield to circumstances of caprice or prejudice. The board proposes that the senior officer of the line present, no matter of what grade, shall preside at mess-table and exercise command over all other members of the mess as far as he may deem necessary to preserve order. Under this law a master or junior lieutenant could command any officer of the staff to leave the dinner table, and in event of restiveness, could have him tried before a court-martial under charge of "disobedience of orders." The fourth section of the bill, or suspension clause, provides, "That when sea officers, civil officers, and engineers shall be required to act together on boards, councils, surveys, or other occasions when their joint views or opinions are to be expressed, and in all messes of officers, the *superior sea officer* present shall preside, whatever may be his relative precedence, and may exercise command over the other members, to direct the course of proceedings, and to preserve order; but, when it can be done without serious inconvenience, the senior sea officer ordered on duties where such joint opinions are required, shall be regularly entitled to take precedence of all the civil officers or engineers who may be ordered to act on such duty."

In a word, the board has carefully provided a legal mode of violating the assimilated rank it has suggested, and places staff officers in the navy relatively to the line, in a position comparable to that of a class of people in the free States of the Union, who are denominated free citizens, though forever denied the right of suffrage and eligibility to office among their fellow tax-payers, under any circumstances whatever. According to the proposed law, a midshipman, or even a coxswain, has precedence of and right to command any medical officer, chaplain, purser, &c., embarked in a boat under his momentary orders. A surgeon or purser attached to a ship, on an official visit to another vessel cannot order a midshipman or coxswain in charge of the boat which brought him to wait; the midshipman may say, I won't remain sir, it is my dinner time. In event of all line officers being killed or disabled, the staff officers, under the proposed law, fall to a level with privates, and might come under the command of a boatswain's mate.

The report, as a whole, labors to establish most of the evils complained of by staff officers. The board has been guided and trammelled by precedents, which, in its appreciation, seem to be stronger than statutes. It is believed that, "Precedents can palliate subsequent errors, but not transmute them into rights, any more than the continuous circulation of a spurious dollar can transmute it into a genuine dollar. Precedents are properly authoritative in courts of law, for they prevent a vacillation in judicial decisions, which, if erroneous, can be corrected by new legislation; but if our national legislature (Congress and President) shall deem itself bound by legislative precedents, no mode exists for correcting errors: 'the salt will, indeed, have lost its savor, and wherewith shall it be salted?' and written constitutions, the American great improvement in government, will lose their quality in rendering principles immutable."

It is presumed members of the profession in the naval service will not willingly accept an assimilated rank in the degrees and terms proposed. It might be just to assemble a board, constituted of staff officers exclusively, to present a scheme of military organization for the navy; such a board would give to the line all the precedence and priority and command necessary to the efficient discharge of duty. The idea of the staff legislating for the line, is not more preposterous than that the line should arrogate to itself exclusively the privilege of legislating for the staff.

It is hoped our readers will take the trouble to make themselves conversant with this question, the final decision of which by the legislature of the Union, must in a manner, settle the standing of the medical profession in the eyes of the nation. It is an interest of every physician, remote though it may be, that the government in all its acts, shall pay due respect to the profession by a just consideration of those members of it employed in the army and navy. To defend them from degradation in the military community, is to defend the standing of the whole profession. Under this view, we recommend that every medical journal throughout the Union take up the subject in defence of a common cause.

